# Evolution of dietary patterns in Flanders: an ecological trend study on best-selling cookbook recipes (2008–2018) and their correlation with household purchases

**DOI:** 10.1186/s12937-024-01004-5

**Published:** 2024-08-24

**Authors:** Viktor Lowie Juliaan Proesmans, Christophe Matthys, Iris Vermeir, Maggie Geuens

**Affiliations:** 1 BE4LIFE, Department of Marketing Innovation and Organisation, Faculty of Economics and Business Administration, Ghent University, Tweekerkenstraat 2, Ghent, 9000 Belgium; 2 Department of Chronic Diseases and Metabolism, Clinical and Experimental Endocrinology, KU Leuven, Herestraat 49, Leuven, 3000 Belgium; 3 Department of Endocrinology, University Hospitals Leuven, Herestraat 49, Leuven, 3000 Belgium

**Keywords:** Cookbooks, Diet, Vegetarian, Nutritive value, Public health, Dietary trends

## Abstract

**Background:**

With rising obesity rates in Western societies, analyzing changes in dietary patterns is paramount. While nutritional surveys have been informative, traditional cookbooks have historically shed light on national cuisines and its changes. Despite the growing popularity of online platforms for food information, cookbooks might still reflect prevalent dietary trends and the diets people follow. This study examined (1) the changes in nutritional content and food group usage in the best-selling cookbooks from 2008 to 2018, and (2) the correlation between the food groups in these cookbooks and dietary patterns (inferred from household purchases) over the same timeframe.

**Methods:**

An exploratory ecological study was conducted on 20 main course recipes of each of the five best-selling cookbooks in Flanders annually between 2008 and 2018. Trends in macronutrients and food group usage in these recipes were analyzed using generalized linear models. Additionally, these trends were compared to household purchase data in Flanders using correlation matrices.

**Results:**

Our results reveal a rising trend towards the use of plant-based ingredients and meat alternatives in cookbooks over the period 2008–2018. There was an increase in the usage of vegetables, nuts & seeds, and cheese. Conversely, there was a decline in the usage of meat, sugar & sweeteners, alcohol, and dairy (all *p*-values < 0.05). In terms of macronutrient content, there was an upswing in carbohydrate, fibre, and sugar levels, while the total fat content showed a decrease (all *p*-values < 0.05). The levels of protein and saturated fat remained consistent over time. Notably, shifts in plant-based and animal-based food group preferences in popular cookbook recipes align with the trends seen in actual household purchases of these food groups (all *p*-values < 0.05).

**Conclusion:**

These findings indicate that cookbook content evolves over time, potentially reflecting shifts in population dietary patterns. Future research is needed to (1) determine any causative link between cookbooks and any causative link between cookbooks and (2) the potential for cookbooks to aid in health promotion.

## Introduction

### Cookbooks and dietary trends

As obesity rates reach epidemic proportions in Western societies, many studies utilize nutritional surveys to analyze changes in the dietary patterns of populations over time [1–3]. One question that arises is whether analyzing recipes in cookbooks could serve this same purpose. While there is limited research on the relationship between cookbooks and dietary patterns in populations, cookbooks have historically played a significant role in establishing national cuisines [4]. Almost every Western-European country boasts a cookbook with a longstanding tradition [5]. Furthermore, previous studies suggest that the content of popular cookbooks from past eras can offer insights into the dietary intake of populations and the nutritional information they received at the time. For example, Mitchell (2008) found that New Zealand cookbooks published between 1940 and 1969 effectively reflected the nutritional guidance communities received during that era [6]. In terms of dietary intake, Eidner et al. (2013) observed a correlation between the rising BMI of the Danish population and the increasing caloric content and portion sizes in the popular Danish cookbook “Mad” from 1909 to 2009 [7]. Similarly, Buisman and Jonkman (2019) noted that the uptick in energy density and protein per kcal in recipes from the Dutch cookbook “Margriet Kookboek” from 1950 to 2010 mirrored trends in the Dutch National Food Consumption Survey for the same period [1]. Such findings suggest that cookbook recipes can indeed serve as valuable tools for identifying trends in dietary patterns.

However, the question remains: can these results be replicated in our current digital society, and can similar findings be observed for other dietary indicators, such as food groups? If so, this could provide insights into potential dietary trends that might emerge in the population in the near future.

### The role of modern cookbooks in society

The role of cookbooks in society has undergone significant changes over the past decades. Firstly, beyond printed media, there has been a surge in the availability of alternative media platforms offering food information. In the past, individuals primarily depended on family, acquaintances, or printed media, but today, they can access an almost limitless array of recipes and food information online [4, 8, 9]. Highlighting the growing importance of online media platforms, a study in 2000 by Caraher et al. found that, aside from parents, cookbooks were the main source for individuals to acquire cooking skills [10]. Notably, this study made no mention of online sources as reference points. Yet, by 2019, a survey by BE4life indicated that people consulted online media as frequently as they did cookbooks for food information, and even more so for actual recipes [11]. Secondly, the nature of the cookbook genre itself has evolved. Beginning in the 1980s, there was an infusion of entertainment elements into cookbooks [8]. The rise of celebrity chefs further propelled this trend towards entertainment, transitioning the narrative towards edutainment in the late 1990s and 2000s [12, 13]. Beyond just instructional content, cookbooks transformed into a hybrid genre, with themes such as family, friends, locality, and enjoyment taking center stage [13, 14]. Elias (2017) notes how celebrity chef cookbooks contain rather complicated recipes, suggesting that readers might not strictly adhere to the instructions. As a result, contemporary cookbooks might be viewed more as sources of inspiration or entertainment rather than practical culinary guidance, which could diminish their influence on actual dietary habits [8]. Lastly, when compared to the previous century, contemporary society witnesses more people dining out and dedicating less time to home cooking, suggesting a potential decline in cookbook reliance [3].

Conversely, there are compelling reasons to believe that cookbooks continue to be valuable tools for identifying dietary trends. First, Proesmans et al. (2022) suggest that beyond immediate circles like family and acquaintances, media icons—specifically celebrity chefs and influencers— are the primary sources of food information in Flanders. Celebrity chefs defined as “professional cooks who have attained a celebrity status through their appearances on media platforms [14]” and influencers defined as “unlicensed native agents of awareness, positioned in traditional and social media to offer emotional support and a pedagogy for self-discovery and well-being [15],” have consistently topped best-seller cookbook lists in regions like Flanders and other Western countries such as the US over the past decade [14]. Their books often encapsulate content derived from other platforms where they are popular like online media and TV shows, and following their advice correlates with shifts in dietary habits [13, 14, 16]. Second, there are instances suggesting a direct link between popular cookbooks and dietary purchases. Notably, following the launch of the prominent local diet book “de voedselzandloper” (translated as ‘the food hourglass’) in Flanders, Belgium, in 2013, there was a 30% surge in oat sales [17].

### Aim

The objective of this study is to determine if contemporary cookbooks remain indicative of dietary trends. Firstly, we will examine the evolution of nutritional content and food group utilization in popular cookbooks from Flanders (Belgium) between 2008 and 2018 (research question 1). Secondly, we will assess whether shifts in food group usage and nutritional content correlate with household food acquisitions (research question 2).

## Method

### Study design

An exploratory ecological study was conducted in Flanders, the Dutch-speaking region of Belgium.

### Data selection

#### Cookbook selection

Using data from the Belgian non-profit organization “boek.be”, the top five best-selling cookbooks for each year from 2008 to 2018 were selected. Cookbooks that exclusively focused on desserts, baking, or one specific type of dish were omitted. If a cookbook was in the top five for two consecutive years, its recipe data was utilized for both years.

#### Cookbook recipe selection

For every chosen cookbook, 20 recipes were selected based on the subsequent criteria:

Inclusion criteria:

- The dish had to be a main course, utilizing at least three distinct food groups, with at least one ingredient requiring heating. The dish could not be categorized as breakfast, lunch, snack, or exclusive for a special event.
- The proportion of fish, meat, and dairy or plant-based recipes in a cookbook had to be retained in the recipe selection. For instance, if the main courses in a cookbook comprised 40% fish recipes and 60% meat recipes, the 20 selected recipes also comprised 40% fish recipes and 60% meat recipes.
- Every other recipe from categories (fish, meat, dairy or plant-based recipes) was chosen until reaching 20 recipes. If category ratios disallowed even distribution, 21 recipes were selected.
- Subcategories within the categories fish, meat and dairy or plant-based recipes were also considered. For instance, if there was one chapter on lamb recipes and one on beef recipes, an equal amount of recipes from each category was included in the analysis.
- If a cookbook lacked 20 eligible recipes, the maximum number meeting the criteria were chosen.

Exclusion criteria.

- Soups were not included, as they are not viewed as full meals in Belgium and other Western European nations [18].

### Household purchase data

The food purchasing habits of average Flemish households from 2008 to 2018 were sourced from household purchase data provided by the professional marketing research agency, GfK [19]. This dataset tracks the annual grocery purchases of 4300 to 6200 Flemish households, detailing specific food categories (e.g., bread & pastry) and individual products within those categories (e.g., yeast), along with the purchased quantities.

### Data preparation

The nutritional content of each ingredient was determined using the Belgian Food Composition Database (Nubel) [20]. If the nutritional content of an ingredient was not available, the USDA food composition database was used to complete missing information [21]. The USDA nutritional database was selected because it is trustworthy and well-established [22]. Furthermore, it is open access, which facilitates data scraping and allows for the automatic linking of food items to their compositions, reducing potential coding errors.

When analyzing the nutritional content of the recipes, the following criteria were used:

- Optional ingredients were incorporated.
- When given ingredient options, the first was selected.
- If provided a range, the smallest quantity was chosen. For example, if the ingredient list stated “4–6 onions,” 4 onions were included in the recipe.
- When portion sizes were defined in (household) measurements (e.g. “a spoon of,” “a cup of,” “a slice of,” “a piece of”), these were converted into grams using the Belgian reference ”Maten en Gewichten” by the Belgian Superior Health Council [23].
- To ensure consistency in the energy content across various recipes, we adjusted the nutritional contributions of total carbohydrates, sugar, total fat, saturated fat, protein, and fiber to align with the energy content of a standard meal, set at 600 kcal [24]. This value was chosen based on the guideline that the energy consumed per eating occasion should not exceed 30% of the daily recommended intake of 2000 kcal [24].
- Additionally, the total weight of each food group present in a meal was recalculated. The formula employed was:
- weight×kcal foodgroup 1 + weight × kcal foodgroup 2 + … + weight×kcal foodgroup x = 600 kcal.

A detailed illustration is provided in Table 1.

**Table 1:** Example of recalculating recipes to 600 kcal

	Original recipe			Recalculation to 600 kcal	
Ingredients	Quantity (gram)	Kcal/100 gram	Calories in recipe (kcal)	Recalculation to 600 (kcal)	Recalculation weight (gram)
Almond flour	100	607	607	301.49	49.67
Eggs	100	151	151	75.00	49.67
Gruyère cheese	100	400	400	198.68	49.67
Passata	200	25	50	24.83	99.34
Total	500		1208	600	248.34

### Data analysis

All ingredients in the recipes were categorized into distinct food groups. This categorization was aligned with the standard food groups found in GfK’s household purchase data. A detailed breakdown of these food groups can be found in Table 2.

**Table 2:** Classification of single foods into specific food groups

Food group	Content	Unit
Alcohol	Alcoholic beverages (e.g. beer, red wine)	Milliliters
Butter and fats	Butter and other animal based fats (e.g. duck fat, suet)	Grams
Cheese	Cheeses (e.g. parmesan, mozzarella)	Grams
Dairy	All dairy with the exclusion of butter and cheese (e.g. milk)	Milliliters
Eggs	Eggs (e.g. chicken or quail egg)	Grams
Fish	Fish and seafood (e.g. salmon, shrimp)	Grams
Fruit	Fruit (e.g. apples, oranges)	Grams
Juice	Fruit juices (e.g. orange juice, lemon juice)	Milliliters
Legumes	Legumes (e.g. beans, chickpeas)	Grams
Meat	All types of meat (e.g. chicken breast, steak)	Grams
Nuts & seeds	Nuts & seeds (e.g. walnut, sunflower seeds)	Grams
Oil	Cooking oils (e.g. olive oil, sunflower oil)	Milliliters
Plant-based meat replacers (planbmr)	Plant-based meat and dairy replacers (e.g. rice milk, seitan, soy milk)	Grams
Potatoes	Potatoes or potato products (e.g. fries)	Grams
Refined grains	Refined grain products (e.g. flour, pasta)	Grams
Sugar and sweeteners	Sweeteners and candies (e.g. sugar, honey, stevia)	Grams
Spices	Spices (e.g. salt, pepper)	Grams
Vegetables	Vegetables and vegetable sauces (e.g. carrots, spinach, tomato sauce)	Grams
Vinegar and sauces	All types of vinegar and premade processed sauces (e.g. vinegar, mayonnaise)	Milliliters
Whole grains	Whole grain products (e.g. whole grain bread)	Grams

For recipes adjusted to contain 600 kcal, we calculated the quantity of each food group used. To draw parallels between the usage of food groups in recipes and actual household purchases, we analyzed purchases from the following food groups: cheese, eggs, butter and fats, refined grains, meat, oil, legumes, nuts & seeds, dairy, vegetables, whole grains, potatoes, and fish. Given that food groups like alcohol and fruit are predominantly consumed independently rather than as meal ingredients, our analysis focused solely on trends where they are utilized.

Using GfK’s household purchase data, we calculated the total annual quantity of each food group purchased on average by a household from 2008 to 2018, as expressed in the units shown in Table 2. When food products within a food group were measured in both milliliters and grams, we converted these into the most commonly used unit (Table 2) using a one-to-one conversion, in accordance with the Belgian Superior Health Council’s reference “Maten en Gewichten” [23].

### Statistical analyses

To determine if the relative quantity of each recipe type (dairy or plant-based, meat, or fish) in the recipe list evolved over time, we employed a chi-square test. An independent samples t-test was utilized to examine shifts in energy density over the studied period. For evaluating trends in the nutritional content and food group usage within recipes, a linear model was chosen. This decision was based on individual univariate residual plots for each macronutrient and food group, which exhibited a linear trend over time [25]. Given that our dataset comprises entries from five distinct cookbooks annually, the independence of the data is influenced by the random effect attributed to the cookbooks [26]. As a result, we applied the generalized linear mixed model (GLMM) approach [27]. The GLMM was executed with a Gamma distribution and a logit link, keeping in mind that our response variables are positive, continuous, and non-additive. For this, the MASS package version 7.3-51.6-1.1804.0 in R was utilized.

For assessing the correlation between the food groups within recipes and contrasting the trends in cookbooks with actual food purchases, we relied on Pearson correlations. For each recipe in popular cookbooks, we adjusted the absolute quantity of ingredients to fit a 600 kcal serving, then measured the total quantity of each food group used in all recipes on an annual basis. For household data, we converted the total quantity of food products purchased by the average household into annual figures. This standardization was necessary to align the time intervals used in measuring cookbook and household data. A correlation was deemed significant if the associated *p*-value was less than 0.05. We evaluated the correlation between the mean annual usage of each food group in cookbooks and the average yearly purchase quantity of each food group among Flemish households. We opted for correlation analysis rather than time-series analysis, as we only have ten different time points after aligning the time intervals of cookbook and household data [28]. For visual representation, plots and heatmaps were crafted using ggplot2 version 3.3.5.

## Results

**Fig. 1:**
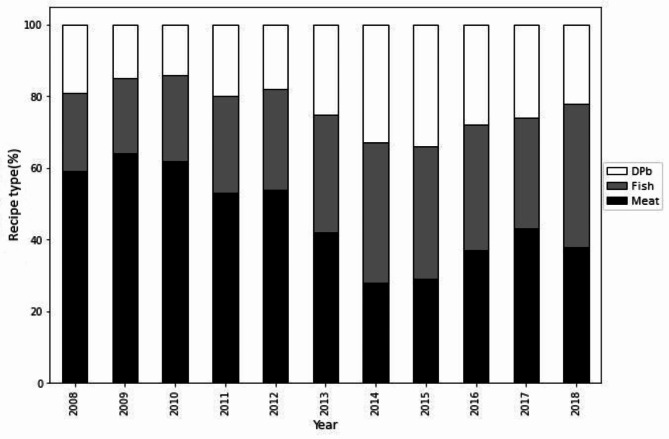
Evolution of recipe types over time. *Recipe types are categorized in meat, DPb (dairy and plant-based) and fish recipes

A total of 38 cookbooks, encompassing 700 recipes, were scrutinized for the study. To investigate how the nutritional content and food group usage in popular cookbooks changed in Flanders (Belgium) over the period 2008–2018 (research question 1), we initiated our analysis with the progression of recipe types, as presented in Fig. 1. There was a pronounced decline in meat-based recipes over the decade, coupled with a notable surge in fish recipes. Dairy or plant-based recipes also displayed a modest uptick. Breaking down the numbers, meat recipes, which constituted 60% of the total in 2008, dwindled to 38% by 2018. On the other hand, fish and dairy or plant-based recipes, which began at 21% and 19% respectively in 2008, expanded to 40% and 22% by 2018 (χ2 (2, *n* = 200) = 25.731, *p* < 0.001).

When analyzing the energy composition (kcal) of recipes in 2008, the division was approximately 53% from total fat, 19% from carbohydrates, and 28% from protein. In 2018 these percentages were 41%, 27%, and 32% respectively (χ2 (2, *n* = 100) = 6.66, *p* = 0.035). Sugar and saturated fat content accounted for 6% and 18% of total energy in 2008 respectively. This was 10% and 12% in 2018.

**Fig. 2:**
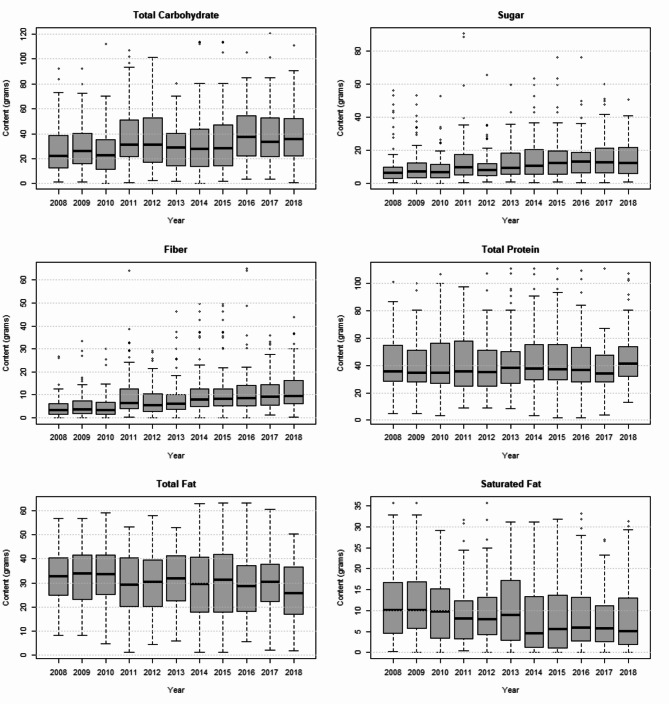
Evolution of macronutrient content in popular cookbook recipes

Figure 2 shows – for a recalculation of each recipe to contain 600 kcal - the evolution of the absolute usage of the macronutrients total fat, saturated fat, total carbohydrate, sugar, fiber and total protein content in the recipes of the most popular cookbooks over the period 2008–2018. Among these, four macronutrients (fiber, carbohydrates, sugar, and total fat) underwent significant alterations. Notably, fiber, carbohydrate, and sugar contents rose markedly (all *p*-values < 0.01), while total fat witnessed a decline (*p* < 0.05). No perceivable fluctuations were observed for protein and saturated fat content. An in-depth exploration of the GLMM findings is accessible in Table 3.

**Table 3:** Trends in macronutrient content among popular cookbook recipes

Variables	Estimates	Standard error	*p*-value
Total fat			
Intercept	31.178	12.442	0.012
Year	-0.014	0.006	0.026*
Saturated fat			
Intercept	54.253	30.163	0.072
Year	-0.026	0.015	0.085
Total carbohydrates			
Intercept	-60.780	20.901	0.004
Year	0.033	0.010	0.002*
Sugars			
Intercept	-91.198	27.879	*p* < 0.001
Year	0.047	0.014	*p* < 0.001*
fibre			
Intercept	-160.380	33.775	*p* < 0.001
Year	0.081	0.017	*p* < 0.001*
Total protein			
Intercept	0.505	17.650	0.977
Year	0.002	0.009	0.856

Notes. Table 3 comprises univariate GLMMs on the evolution in macronutrients over a 10-year period. Cookbooks are used as a random factor

*The content changed significantly over 10 years, *p* < 0.05

**Fig. 3:**
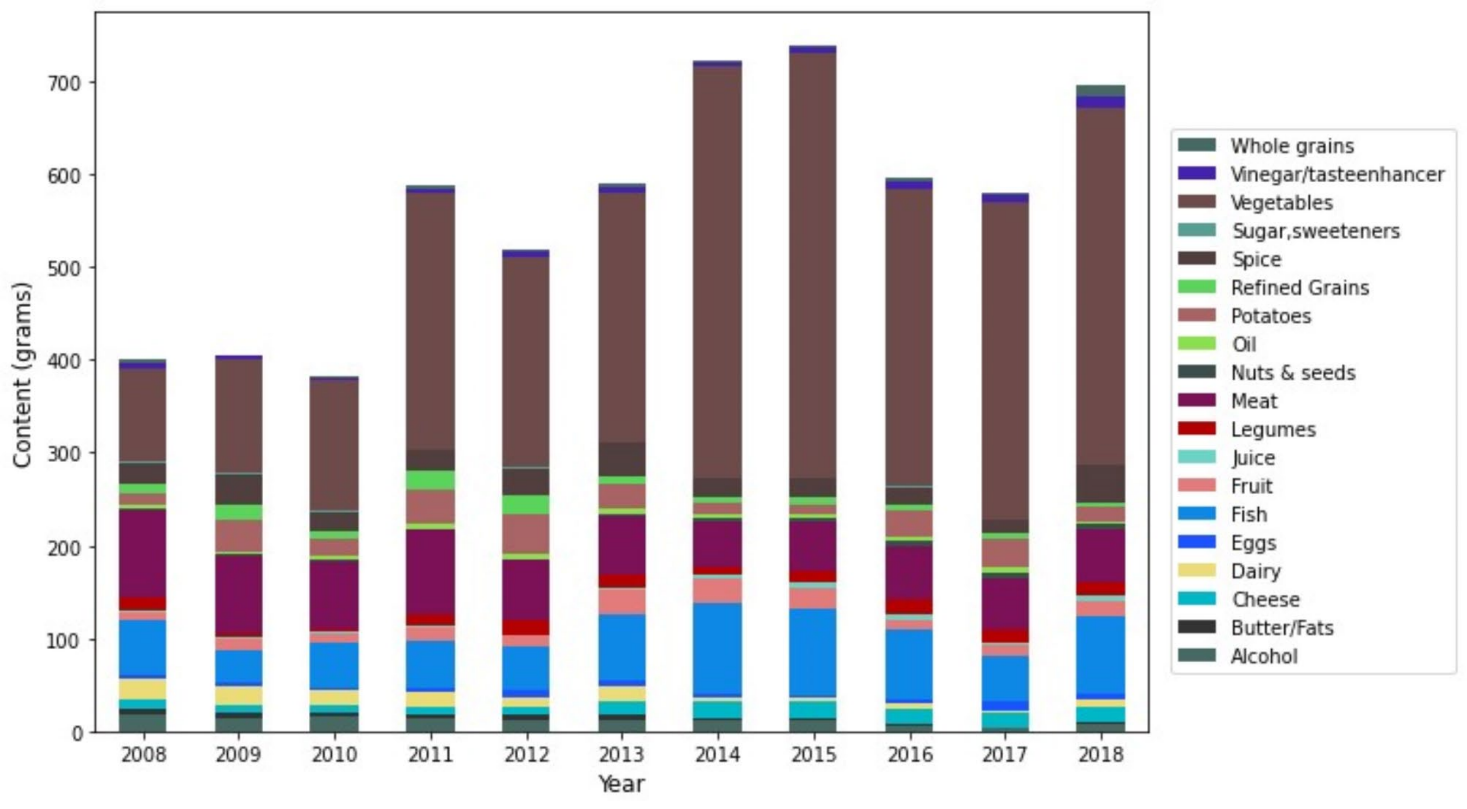
Food group usage in popular recipes. *All quantities were converted into grams

Figure 3 illustrates the quantity – adjusted to represent each recipe as containing 600 kcal – of each food group utilized between 2008 and 2018. Recipes became less energy-dense over this period; the average recipe in 2008 weighed 403 g (1.49 kcal/g), while by 2018, it had increased to 737 g (0.814 kcal/g) (*p* < 0.001).

Regarding the evolution in food group usage, there was an observed increase in the use of vegetables, nuts & seeds, and cheese over time (all *p*-values < 0.01). In contrast, there was a decrease in the use of meat, dairy, sugar & sweeteners, and alcohol (all *p*-values < 0.01) (Table 4).

**Table 4:** Trends in food group usage in cookbook recipes over the period 2008–2018

Variables	Estimates	Standard error	*p*-value
Alcohol			
Intercept	146.378	40.116	0.001
Year	-0.074	0.020	0.001*
Butter and fats			
Intercept	26.869	19.124	0.160
Year	-0.014	0.009	0.128
Cheese			
Intercept	-101.933	32.264	0.002
Year	0.050	0.016	0.002*
Dairy			
Intercept	141.313	41.526	0.001
Year	-0.071	0.021	0.001*
Eggs			
Intercept	-52.032	29.596	0.079
Year	0.025	0.014	0.091
Fish			
Intercept	-80.960	58.176	0.164
Year	0.040	0.029	0.166
Fruit			
Intercept	-25.840	49.163	0.599
Year	0.012	0.024	0.622
Juice			
Intercept	-40.498	20.397	0.047
Year	0.019	0.010	0.060
Legumes			
Intercept	-58.097	50.729	0.252
Year	0.028	0.025	0.266
Meat			
Intercept	144.456	34.059	0.001
Year	-0.072	0.017	0.001*
Nuts & seeds			
Intercept	-50.466	17.126	0.003
Year	0.024	0.009	0.005*
Oil			
Intercept	30.711	21.176	0.147
Year	-0.016	0.011	0.119
Plantbmr			
Intercept	-32.253	33.298	0.333
Year	0.015	0.017	0.365
Potatoes			
Intercept	65.313	74.330	0.380
Year	-0.033	0.037	0.370
Refined grains			
Intercept	74.766	46.699	0.110
Year	-0.038	0.023	0.102
Sugar and sweeteners			
Intercept	17.873	7.446	0.017
Year	-0.010	0.004	0.007*
Spices			
Intercept	53.057	84.758	0.532
Year	-0.027	0.042	0.520
Vegetables			
Intercept	-178.032	46.707	0.001
Year	0.089	0.023	0.001*
Vinegar			
Intercept	-50.535	32.081	0.116
Year	0.024	0.016	0.131
Whole grains			
Intercept	-51.317	27.500	0.062
Year	0.024	0.014	0.074

Notes. Table 4 comprises a GLMM on the evolution in macronutrients in a 10-year period. The cookbooks in which the recipes were included are used as a random factor

*The content has significantly changed over 10 years, *p* < 0.05

**Fig. 4:**
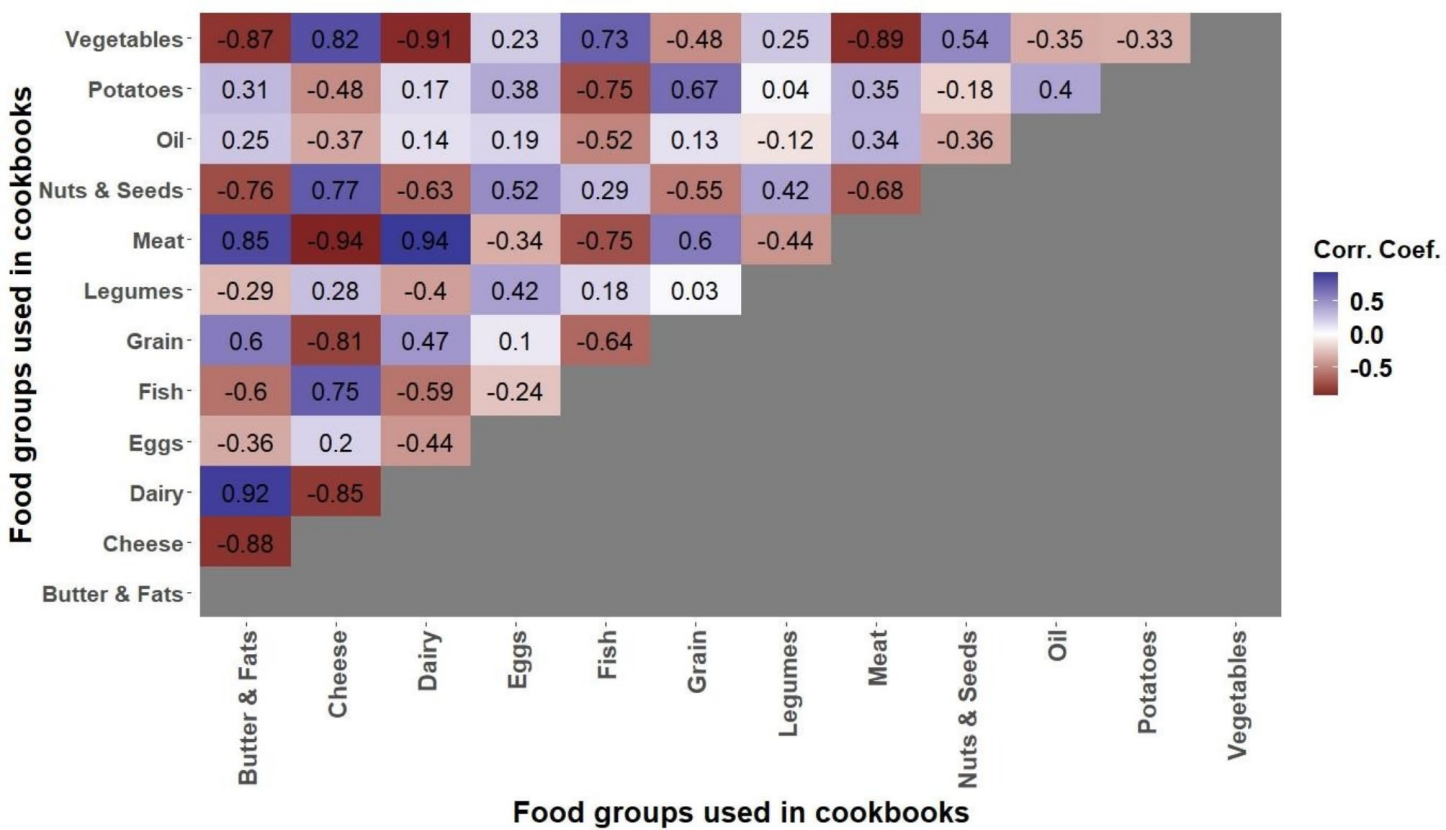
Correlation matrix of food groups within cookbook recipes for the period 2008–2018

To gain a clearer understanding of the overarching trends in food group usage within cookbooks, Fig. 4 illustrates the correlations among the different food groups.

Figure 4 illustrates that the inclusion of vegetables as an ingredient in a recipe is negatively correlated with the use of meat (*r* = -0.89, *p* < 0.001), butter & fats (*r* = -0.87, *p* < 0.001), and dairy (*r* = -0.91, *p* < 0.001). Conversely, there is a positive correlation with fish (*r* = 0.73, *p* = 0.01) and cheese (*r* = 0.82, *p* = 0.002). Similarly, the usage of meat and dairy in a recipe is positively correlated (*r* = 0.94, *p* < 0.001) as is their correlation with butter & fats (*r* = 0.85 & *r* = 0.92, both *p*-values < 0.001). These ingredients negatively correlate with cheese (*r* = -0.94 & *r* = -0.85, both *p*-values < 0.001), nuts & seeds (*r* = -0.68 & *r* = -0.63, *p* = 0.022 & *p* = 0.039 respectively), and for meat, with fish (*r* = -0.75, *p* = 0.008). The usage of fish positively correlates with vegetables (*r* = 0.73, *p* = 0.01) and cheese (*r* = 0.75, *p* = 0.008) and is negatively correlated with meat (*r* = -0.75, *p* = 0.008), grains (*r* = -0.64, *p* = 0.03), and potatoes (*r* = -0.75, *p* = 0.008).

**Fig. 5:**
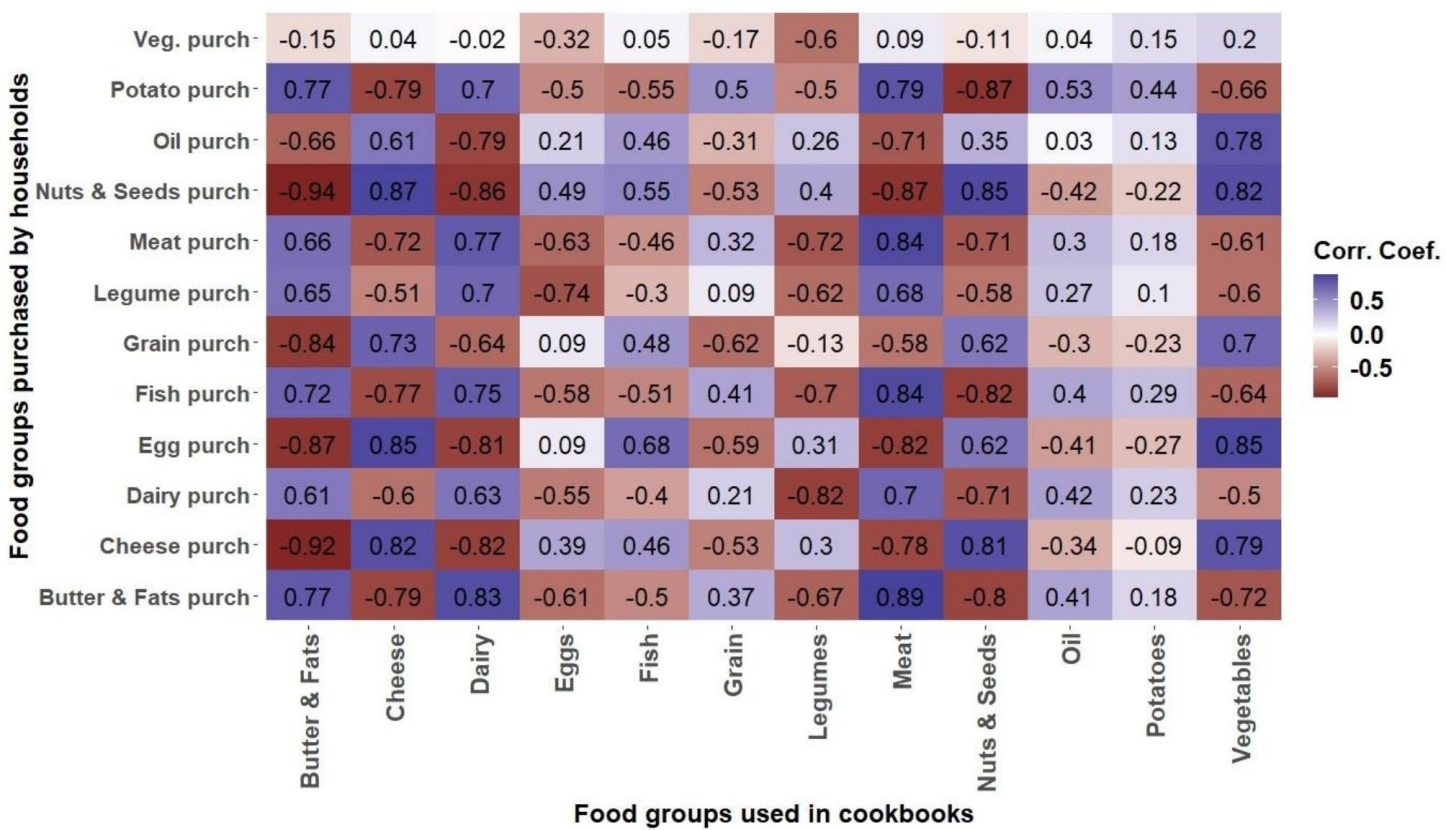
Correlation matrix between household purchases and recipe content for the period 2008–2018

To determine if changes in food group usage and nutritional content align with household food purchases (research question 2), Fig. 5 presents the correlations between food group preferences in recipes and actual household food purchases.

The use of vegetables as ingredients in a recipe correlates positively with the purchases of oil (*r* = 0.78, *p* = 0.005), eggs (*r* = 0.85, *p* = 0.001), grains (*r* = 0.70, *p* = 0.018), cheese (*r* = 0.79, *p* = 0.004), and nuts & seeds (*r* = 0.82, *p* = 0.002). Conversely, it negatively correlates with the purchases of legumes (*r* = -0.61, *p* = 0.049), meat (*r* = -0.61, *p* = 0.047), fish (*r* = -0.64, *p* = 0.032), butter & fats (*r* = -0.72, *p* = 0.012), and potatoes (*r* = -0.66, *p* = 0.026).

The use of nuts & seeds in a recipe has a positive correlation with the purchases of eggs (*r* = 0.62, *p* = 0.043), grains (*r* = 0.62, *p* = 0.043), cheese (*r* = 0.81, *p* = 0.002), and nuts & seeds (*r* = 0.81, *p* = 0.002). On the other hand, it negatively correlates with purchases of meat (*r* = -0.71, *p* = 0.015), fish (*r* = -0.82, *p* = 0.002), butter & fats (*r* = -0.80, *p* = 0.003), dairy (*r* = -0.71, *p* = 0.014), and potatoes (*r* = -0.87, *p* < 0.001).

Including meat and dairy as ingredients in recipes correlates positively with the purchases of meat (*r* = 0.84 & *r* = 0.77, *p* = 0.001 & *p* = 0.006), legumes (*r* = 0.68 & *r* = 0.70, *p* = 0.023 & *p* = 0.016), fish (*r* = 0.84 & *r* = 0.75, *p* = 0.001 & *p* = 0.007), butter & fats (*r* = 0.89 & *r* = 0.83, *p* < 0.001 & *p* = 0.002), dairy (*r* = 0.70 & *r* = 0.63, *p* = 0.016 & *p* = 0.038), and potatoes (*r* = 0.79 & *r* = 0.70, *p* = 0.004 & *p* = 0.017). These ingredients negatively correlate with purchases of nuts & seeds (*r* = -0.87 & *r* = -0.86, both *p*-values = 0.001), cheese (*r* = -0.78 & *r* = -0.82, *p* = 0.005 & *p* = 0.002), oil (*r* = -0.71 & *r* = -0.79, *p* = 0.015 & *p* = 0.004), and eggs (*r* = -0.82 & *r* = -0.81, both *p*-values = 0.002). For dairy, there is a negative correlation with grain purchases (*r* = -0.64, *p* = 0.035).

Interestingly, within specific food groups, no significant correlation was observed between the purchase and usage in recipes of vegetables (*r* = 0.021, *p* = 0.55), potatoes (*r* = 0.044, *p* = 0.18), fish (*r* = -0.51, *p* = 0.11), oil (*r* = 0.03, *p* = 0.93), and eggs (*r* = 0.09, *p* = 0.79). A negative correlation was noted for legumes (*r* = -0.62, *p* = 0.042) and grains (*r* = -0.62, *p* = 0.043) in terms of their purchase and use within recipes.

## Discussion

This study examined the evolution of recipes in popular cookbooks from 2008 to 2018 and assessed how the ingredients in these recipes correspond with food purchases among Flemish households. These cookbooks revealed a noticeable trend towards fewer meat recipes and a rise in fish and dairy or plant-based recipes. Moreover, there is an increasing preference for plant-based ingredients like vegetables, accompanied by a decline in meat and dairy use. On the macronutrient front, recipes reflected reduced total fat content while showcasing an increase in carbohydrates, sugar, and fiber. Notably, our findings highlight a correlation between food groups in cookbook recipes and the purchasing habits of Flemish households.

A traditional Flemish main course typically includes meat or fish, vegetables, potatoes and often a sauce [5, 29]. Classic Flemish cookbooks and dietary habits traditionally emphasized meat as a central constituent of the Flemish diet [5]. The recent shift towards fewer meat-based recipes in modern cookbooks is noteworthy but aligns with international and Flemish government dietary guidelines that promote plant-based diets and sustainable food consumption [30, 31]. Reynolds (2022) also observed a global trend where omnivorous cookbook recipes are leaning more plant-based [32]. Consistent with this gradual pivot towards plant-centric recipes, a growing segment of the population in Belgium and other Western countries is diminishing their meat consumption, gravitating towards vegetarian or flexitarian diets [33, 34].

In terms of macronutrient composition, our study observed a decrease in total fat content in recipes between 2010 and 2018, with an upward trajectory for total carbohydrates, sugar, and fiber. However, total protein and saturated fat contents remained consistent. Considering the Flemish population’s average intake leans towards excessive fat and insufficient complex carbohydrates, the trends in cookbook content seem beneficial [35]. A plausible rationale for the spike in carbohydrate and fiber content relative to fat is the broader acceptance of plant-based diets. Incorporating more vegetables typically augments the carbohydrate and fiber content, while minimizing meat and dairy reduces fat content [36]. Interestingly, the sugar content rose despite a curbed use of added sugars and sweeteners. The extra sugar, therefore, is likely not added sugars, but natural sugar from food groups like fruit or vegetables, which besides sugar also contain vitamins, minerals and antioxidants [37, 38]. However, our current methodology doesn’t allow us to verify this definitively.

The recipes from 2018 remain notably high in fat and protein, reflecting the influence of influencers who dominated the Flemish cookbook charts between 2013 and 2018, championing low-carbohydrate diets [39]. The Belgian Superior Health Council advises a daily energy intake comprised of 50–55% carbohydrates, 30–35% fat, and 15% protein. However, cookbook recipes from 2018 had 27% carbohydrates, 41% fat, and 32% protein [35]. Despite a sugar increase, these recipes conform to dietary guidelines, with sugar constituting 10% of total calories. Saturated fat accounted for 12% of total calories, slightly above the recommended 10%.

These findings echo previous studies that found recipes from celebrity chefs, online platforms, and healthy eating blogs to be high in fat, saturated fat, and protein [18, 40–42]. The influence of globalization on cookbook discourses and food media phenomena like the rise of celebrity chef or influencers, also seems to manifest in cookbook recipes [13]. The macronutrient trends in our study resonate with those from the Belgian National Food Consumption Survey (NFCS) conducted in 2004 and 2014 [43]. Similar to our study, the NFCS reported a reduced total fat intake and increased carbohydrate intake from vegetables by the Flemish population, with consistent total protein consumption. However, unlike our findings, the NFCS did not record significant shifts in total carbohydrate consumption.

The shift in ingredients featured in cookbook recipes further emphasizes the global trend towards plant-based diets. The notable rise in the use of vegetables, nuts & seeds, and cheese, contrasted by a decrease in meat, dairy, sugar, sweeteners, and alcohol, aligns well with international dietary recommendations which advocate for increased consumption of vegetables and nuts & seeds, and a reduction in meat intake [44, 45]. While the Belgian Superior Health Council recognizes dairy as a dietary staple, the evident rise in cheese recipes and the simultaneous decline in other dairy product usage make it ambiguous if the overall dairy consumption aligns with the council’s guidelines [44]. It is therefore, unclear whether the usage of total dairy have become more in line with the guidelines of the Superior Health council. Some studies do suggest replacing dairy with nuts or other plant-based sources would decrease cardiovascular diseases and all-cause mortality. This observed negative correlation between the usage of nuts & seeds and dairy in recipes might then, indeed have beneficial implication [46].

The observed decline in meat and dairy use in recipes has an inverse correlation with the use of vegetables and cheese. Conversely, there is a positive association between vegetable use and both fish and cheese use. This can be attributed to the rising popularity of pescatarian, flexitarian, and vegetarian diets that often utilize fish and cheese as substitutes for meat [47, 48]. While total protein content remains unchanged, the general decrease in meat and dairy usage compared to nuts & seeds and vegetables usage implicates a move towards plant-based protein sources. As it is harder to fulfill the protein requirements with plant-based sources, enough variety remains important [33]. Yet, past research has underscored the health benefits of a higher intake of plant-derived proteins over their animal-based counterparts, highlighting a reduced risk of cardiovascular diseases and overall mortality [49]. Comparing these trends to the Belgian food consumption, the FNCS recorded no significant alterations in meat and vegetable intake from 2004 to 2014. However, the non-profit EVA points to a downward trajectory in meat consumption in subsequent years [50]. The reduction in meat purchases resonates with the prevailing skepticism in the scientific community towards red and processed meats, driven by environmental and health apprehensions [51, 52]. This sentiment seemingly reverberates in both consumer purchasing behaviors and the narratives of mainstream media .

The observed correlations between ingredients featured in cookbook recipes and household purchases illuminate a significant insight into the relationship between media representation and consumer behavior. Notably, there is a pronounced alignment between the rising prominence of plant-based food groups and cheese in recipes and an uptick in household purchases of these items. This concurrence underscores a decrease in meat-centric recipes, leading to households increasing their purchases of alternatives like cheese, oils, eggs, and nuts & seeds. Meanwhile, the amplified vegetable presence in recipes is accompanied by increased purchases of cheese, grains, and nuts & seeds, indicative of a pivot towards dietary guidelines [53]. Fish usage in cookbooks was the only food group that showed a negative correlation with meat usage. However, it displayed a positive relationship with meat in household purchases. This indicates that an increase in fish recipes in cookbooks, as part of a move to reduce meat consumption, does not necessarily lead to more fish purchases. Moreover, as the rise in fish recipes coincides with a trend towards plant-based recipes, individuals might become more aware of their consumption of animal-based products, leading them to decrease their fish purchases. Supporting this idea, fish usage in recipes has a negative correlation with fish household purchases, though it is not statistically significant.

When examining specific food groups, no correlation was found between their usage in cookbooks and the purchases of vegetables, potatoes, and eggs. This lack of correlation is likely because few recipes primarily focus on these ingredients. With oils, the discrepancy might arise because authors do not consistently report the quantity used, potentially introducing bias to the results. A notable negative correlation was observed for the food groups grains and legumes.

As a typical Flemish breakfast comprises cereals or sandwiches with a variety of toppings, including both sweet and savory options, and a typical Flemish lunch consists of sandwiches with cheese or ham, the prevalent use of grain products like bread and cereals in these meals might explain the weaker correlation between the usage of grain products in main course recipes in cookbooks and its occurrence in household purchases [29]. Legumes, on the other hand, are not typically used as a primary protein source in traditional Flemish diets but rather as a vegetable [54]. This might account for their lack of correlation.

Overall, the observed correlations and consistent trends between cookbook recipes and household purchases underscore an association between media consumption and dietary patterns. Boylan et al. (2012) highlighted that media serve as the primary source of food information for most people. Similarly, Proesmans et al. (2022) identified celebrity chefs as the foremost source of food information, with family and influencers following closely behind. These correlations emphasize the viability of cookbook recipe content as a barometer for dietary trends within the general populace, a notion previously posited by Buisman & Jonkman in 2019 [1].

This study presents several limitations that warrant discussion. Firstly, there are subtle discrepancies between the nutritional data procured from the NUBEL and the USDA nutritional databases. This is because ingredients in American food products can vary from those in Belgian products. For instance, cereal grain products in the US are mandated to be enriched with synthetic folic acid since 1997, which may lead to slight differences when compared to Belgian products [55]. Secondly, in our analysis of cookbook recipes, we consistently chose the lower quantity when given a range for an ingredient. Similarly, when presented with multiple ingredient options, we always opted for the first. Such choices might introduce a minor bias in the results. Thirdly, as the literature suggests, some degree of caution is warranted when interpreting food purchase data as a reflection of dietary intake [56]. For instance, unlike food balance sheets, food purchase data does not account for out of home consumption. Given the increasing importance of eating out of home, this omission is a significant limitation in our data [57]. Furthermore, there are differences between what households buy, what they actually consume, and what they use as a main ingredient in a dish. We also do not know how people prepare their meals or whether they follow cookbook recipes exactly. Lastly, a time lag might exist between the acquisition of a cookbook and subsequent household purchases. Given that our data is annual and comprises only ten time points, we were unable to conduct time-series analysis to establish possible lagged effects, as such analyses require a minimum of forty observations [28].

Prior research exploring the relationship between dietary intake and cookbook content has predominantly focused on cross-sectional data or on the content of a singular cookbook [1, 7]. Such approaches are likely inadequate to fully capture evolving trends. Additionally, our annual data on household purchases sheds new light on the highly discussed link between recipe content in mainstream media and dietary patterns, as gauged by household purchases in our study. Given the increasing reliance on media as the primary source of food information, comprehending media’s role in disseminating health and food knowledge becomes imperative for effective health communication and promotion [11]. To ascertain the presence of causal effects, future studies adopting an experimental design are necessary.

## Conclusion

This study aimed to investigate whether modern cookbooks remain indicative of contemporary dietary trends. We examined (1) the evolution of nutritional content and food group usage in popular cookbooks in Flanders from 2008 to 2018, and (2) how changes in these factors correlate with household food purchases. Results indicate a decline in meat recipes in cookbooks over time, with a rise in pescatarian and a slight increase in dairy or plant-based recipes. Generally, the nutritional content in cookbooks has shifted to align more closely with dietary guidelines. This is evident in the increased emphasis on vegetables, nuts & seeds, and cheese as replacements for meat and dairy. Additionally, recipes now contain less total fat but more fiber, sugar, and carbohydrates. Crucially, these shifts in cookbook dietary trends correspond with food purchases in Flemish households, highlighting a relationship between cookbook content and dietary habits. Thus, this study suggests that the recipe content in cookbooks remains a valuable tool for identifying current dietary trends. The potential for cookbooks to directly influence dietary choices is a compelling avenue for future research. Consequently, we advocate for additional studies to explore the extent to which cookbooks can be utilized for health promotion.

## Data Availability

The data that support the findings of this study are available from GfK but restrictions apply to the availability of these data, which were used under license for the current study, and so are not publicly available. Data are however available from the authors upon reasonable request and with permission of GfK.
