# Correction: Evolution of dietary patterns in Flanders: an ecological trend study on best-selling cookbook recipes (2008–2018) and their correlation with household purchases

**DOI:** 10.1186/s12937-024-01008-1

**Published:** 2024-09-12

**Authors:** Viktor Lowie Juliaan Proesmans, Christophe Matthys, Iris Vermeir, Maggie Geuens

**Affiliations:** 1 BE4LIFE, Department of Marketing Innovation and Organisation, Faculty of Economics and Business Administration, Ghent University, Tweekerkenstraat 2, Ghent, 9000 Belgium; 2 Department of Chronic Diseases and Metabolism, Clinical and Experimental Endocrinology, KU Leuven, Herestraat 49, Leuven, 3000 Belgium; 3 Department of Endocrinology, University Hospitals Leuven, Herestraat 49, Leuven, 3000 Belgium

**Correction: Nutr J 23, 99 (2024)**

10.1186/s12937-024-01004-5

Following publication of the original article [1], the author reported that in the “Conclusion” section of the “Abstract”, the text “These findings indicate that cookbook content evolves over time, potentially reflecting shifts in population dietary patterns. Future research is needed to determine (Buisman ME, Jonkman J. Dietary trends from 1950 to 2010: a Dutch cookbook analysis. J Nutr Sci [Internet]. 2019 ed [cited 2022 Apr 19];8. https://www.cambridge.org/core/journals/journal-of-nutritional-science/article/dietary-trends-from-1950-to-2010-a-dutch-cookbook-analysis/ AB281ADE0F09FF8F518B8AC4A2A5BEA8#supplementary-materials) any causative link between cookbooks and dietary habits, and (Ashwell M, Barlow S, Gibson S, Harris C. National Diet and Nutrition Surveys: the British experience. Public Health Nutr. 2006;9(4):523–30.) the potential for cookbooks to aid in health promotion”.

Should only read as:

“These findings indicate that cookbook content evolves over time, potentially reflecting shifts in population dietary patterns. Future research is needed to (1) determine any causative link between cookbooks and any causative link between cookbooks and (2) the potential for cookbooks to aid in health promotion”.

The original article has been updated.
